# Comparative Effectiveness of Methotrexate versus Methylprednisolone in Treatment Naïve Pulmonary Sarcoidosis Patients

**DOI:** 10.3390/diagnostics11081401

**Published:** 2021-08-03

**Authors:** Jotam G. Pasipanodya

**Affiliations:** Independent Researcher, 14830 Venture Drive, Dallas, TX 75234, USA; jpasipanodya@hotmail.com

**Keywords:** sarcoidosis, machine learning, treatment outcomes

## Abstract

Among those who study granulomatous diseases, sarcoidosis is of tremendous interest, not only because its cause is unknown, but also because it is still as much an enigma today as it was 150 years ago when Jonathan Hutchinson first described the cutaneous form of the disease as “livid papillary psoriasis”. This piece editorializes a comparative effectiveness study of methotrexate versus methylprednisolone in treatment naïve pulmonary sarcoidosis patients for CT-guided clinical responses and drug-related adverse events.

Sarcoidosis is a multisystem, noncaseous granulomatous disease that may affect any organ, suggesting a wide spectrum in disease presentation, symptomatology, and severity. Therefore, there is even greater variability in the individuals’ need for pharmacological therapy as well as for other clinical interventions. The disease commonly involves the lungs and less commonly the peripheral lymph nodes, skin, eyes, and the liver. For sarcoidosis diagnosis, histologic confirmation of granulomatous inflammation from at least two organs is required [1,2,3]. Since sarcoidosis has no known causes, this further complicates basic sciences’ search for definitive biomarkers, diagnostics and therapeutics because of unavailability of viable in vitro and in vivo models. The first official American Thoracic Society (ATS) guidelines acknowledges that sarcoidosis diagnosis is “never secure” [2]—but we would also add, “neither are the pharmacotherapeutics”. The currently recommended pharmacologic interventions are empiric, have an unsubstantiated rank order and hardly contain much in terms of radars to track disease progression, predict disease relapse or support the use of one therapy over the other. Perhaps clearer pictures will emerge with ongoing better designed randomized controlled trials with clinically meaningful surrogate pharmacodynamic endpoints [4], including of antimycobacterial therapy (levofloxacin, ethambutol, rifampin and azithromycin, also called CLEAR regimen) in phase II (NCT02024555) and a variety of other drugs, such as host-directed therapies in different stages of clinical trials [3]. Of the 257 sarcoidosis trials registered at Clinicaltrials.gov [as of 29 July 2021], 49 (19%) were actively recruiting patients. Nevertheless, for goals of therapy that seek to prevent or minimize end-organ damage, relieve symptoms and improve health-related quality of life (HRQoL) measures, the following adage for first-line sarcoidosis therapy “…are there acceptable substitutes for systemic corticosteroids?” is still true and relevant today, as it was in 1975, when it was first asked at an international symposium in New York, and thereafter at numerous other times [1,5,6,7,8,9].

Sarcoidosis accounts for almost $10 billion annually in healthcare-related spending [10], has an annual incidence and point prevalence of 8.3 and 59.8 per 100,000 population, respectively, [rates are twice as high among blacks] in the US [11]. While considerable variability in geo-spatiotemporal factors, particularly with regards to overall disease burden (incidence/prevalence/mortality) by sex is still unravelling [12,13,14,15,16], there is no doubt that sarcoidosis significantly contributes to global diseases burden and is a source of constant pain and anguish to the brave individuals living with the disease. Notably, among those who study granulomatous diseases, sarcoidosis is of tremendous interest, not only because of the unknown cause, but also because it is still as much an enigma today as it was 150 years ago when Jonathan Hutchinson first described the cutaneous form of the disease as “livid papillary psoriasis” [17].

In the current issue of the journal, Gavrysyuk et al. use real-world evidence [18], obtained from an observational study of 143 newly diagnosed pulmonary sarcoidosis patients, including 45 (31.5%) with extrapulmonary manifestation and 29 (20%) with moderate/severe disease, to demonstrate same rates of treatment completion and side-effects in head-to-head comparison of methylprednisolone (MP) and methotrexate (MTX). In their study, 97 (68%) enrolled patients were assigned to MP and the remaining 46 patients who could not tolerate corticosteroids for a variety of reasons, such as diabetes mellitus and obesity, were assigned to MTX. The proportion of patients with severe disease was equally balanced between the two groups, with abnormal pulmonary functions test observed in 17/97 (18%) and 12/46 (26%), respectively, *p* = 0.235. The authors reported that treatment completion reviewed with normalization of computed tomography (CT) data with MP was in 68 (70%) patients, while that with MXT was in 29 (63%) patients, *p* = 0.399; adverse events were 5 (5%) and 5 (11%), respectively, *p* = 0.211. Interestingly, the time-to-remission was 18% faster with the MXT arm (mean ± standard deviation [SD] in months 12.7 ± 3 versus 10.8 ± 2.7, *p* < 0.05). What is noteworthy too is that most of the gains in the steroid-sparing regimens were realized in patients who received higher doses of MXT at 15 mg/week, while 9/10 failures (those who failed to demonstrate treatment effectiveness on radiological examination) were in those who received lower dose: 10 mg/week of MXT. In the words of the authors, “MXT monotherapy did not significantly differ with MP monotherapy, in effectiveness and in serious side effects” [18]. Indeed, the group from Kyiv must be commended for their hard work, clinical competency and the timely release of these data that will certainly enhance the care of sarcoidosis patients.

There are some shortcomings in the study, however. For example, time-to-event analyses perhaps with propensity scoring to balance baseline risk factors would be a more elegant approach to tease out individual treatment effect sizes. Alternatively, novel pharmacometrics and agnostic data-science driven approaches, such as stochastic gradient boosting with regression trees, as was shown recently for the sister caseating granulomatous disease tuberculosis [19,20,21], helps with data reduction leaving factors driving treatment response variance to be fully examined. Such approaches, would give the answers to the following important questions: Who stands to benefit most with steroid-sparing? When do we exhaust the benefits of first-line steroid therapy? How much of the variance in outcomes is explained by other covariates, including initial and follow-up spirometry, radiology and blood readouts (using Friedman’s H-index [22])? The optimal means to track and quantify treatment responses for sarcoidosis are still unclear [1,2,3]. However, in addition to serial imaging (high-resolution CT scans), serial changes in symptoms, physiologic and pulmonary function, as well as other HRQoL measures are required and necessary when tracking disease progression. Gavrysyuk et al. reported symptoms and CT imaging only [18]. All clinical observations are important, and they give us valuable insights about a disease we still know little about during therapy. Therefore, those data and any other additional observational data must be quantitatively examined in better ways. The following more basic and generic questions still need answers: what ranked factors and patterns predict therapeutic response? Identifying linear correlations would be interesting and rare but picking nonlinear and more complicated relationships is more likely. Meanwhile, 16-weeks of the CLEAR regimen (NCT02024555) failed to improve forced vital capacity (−1.1% versus 0.02%, *p* = 0.64), radiography, HRQoL or the 6-min walk distance when compared to placebo [23]. Thus, the work to better describe the relationships between pharmacotherapeutics and patients’ response; such as drug dose responses and identifying optimal exposures associated with patients’ outcomes, as demonstrated with MXT in the study by Gavrysyuk et al. [18], still lies ahead of us.

These critiques do not in any way diminish the findings by Gavrysyuk et al. [18]. What is more reassuring is that there are similar findings reported by the same group previously, and related findings by others [7,8,9]. For example, prolonged use of low dose corticosteroids with methotrexate was associated with preserved ejection fraction and fewer adverse events in patients with cardiac sarcoidosis lesions, suggesting that the benefits of methotrexate as first-line extends beyond pulmonary disease [7,8,9]. Together, these studies suggest that the findings might be reproducible in different populations and in different geographic settings. Increased sustained funding for larger international and collaborative studies is much needed for sarcoidosis.

## Data Availability

Study does not report any data.

## References

[1] Statement on Sarcoidosis (1999). Joint Statement of the American Thoracic Society (ATS), the European Respiratory Society (ERS) and the World Association of Sarcoidosis and Other Granulomatous Disorders (WASOG) adopted by the ATS Board of Directors and by the ERS Executive Committee, February 1999. Am. J. Respir. Crit. Care Med..

[2] Crouser E.D., Maier L.A., Wilson K.C., Bonham C.A., Morgenthau A.S., Patterson K.C., Abston E., Bernstein R.C., Blankstein R., Chen E.S. (2020). Diagnosis and Detection of Sarcoidosis. An Official American Thoracic Society Clinical Practice Guideline. Am. J. Respir. Crit. Care Med..

[3] Spagnolo P., Rossi G., Trisolini R., Sverzellati N., Baughman R.P., Wells A.U. (2018). Pulmonary sarcoidosis. Lancet Respir. Med..

[4] Kahlmann V., Janssen Bonás M., Moor C.C., van Moorsel C.H.M., Kool M., Kraaijvanger R., Grutters J.C., Overgaauw M., Veltkamp M., Wijsenbeek M.S. (2020). Design of a randomized controlled trial to evaluate effectiveness of methotrexate versus prednisone as first-line treatment for pulmonary sarcoidosis: The PREDMETH study. BMC Pulm. Med..

[5] Miller A. (2002). Of time and experience: Sarcoidosis revisited. Chest.

[6] Millward K., Fiddler C.A., Thillai M. (2021). Update on sarcoidosis guidelines. Curr. Opin. Pulm. Med..

[7] Fang C., Zhang Q., Wang N., Jing X., Xu Z. (2019). Effectiveness and tolerability of methotrexate in pulmonary sarcoidosis: A single center real-world study. Sarcoidosis Vasc. Diffus. Lung. Dis..

[8] Nagai S., Yokomatsu T., Tanizawa K., Ikezoe K., Handa T., Ito Y., Ogino S., Izumi T. (2014). Treatment with methotrexate and low-dose corticosteroids in sarcoidosis patients with cardiac lesions. Intern. Med..

[9] Gavrysyuk V., Merenkova E., Gumeniuk G., Gumeniuk M., Dziublyk Y. (2018). Effectiveness and safety of methotrexate monotherapy in patients with pulmonary sarcoidosis. Georgian Med. News.

[10] Rice J.B., White A., Lopez A., Conway A., Wagh A., Nelson W.W., Philbin M., Wan G.J. (2017). Economic burden of sarcoidosis in a commercially-insured population in the United States. J. Med. Econ..

[11] Baughman R.P., Field S., Costabel U., Crystal R.G., Culver D.A., Drent M., Judson M.A., Wolff G. (2016). Sarcoidosis in America. Analysis Based on Health Care Use. Ann. Am. Thorac. Soc..

[12] Cozier Y.C. (2016). Assessing the worldwide epidemiology of sarcoidosis: Challenges and future directions. Eur. Respir. J..

[13] Arkema E.V., Grunewald J., Kullberg S., Eklund A., Askling J. (2016). Sarcoidosis incidence and prevalence: A nationwide register-based assessment in Sweden. Eur. Respir. J..

[14] Kearney G.D., Obi O.N., Maddipati V., Mohan A., Malur A., Carter J.C., Thomassen M.J. (2019). Sarcoidosis deaths in the United States: 1999–2016. Respir. Med..

[15] Rossides M., Kullberg S., Eklund A., Grunewald J., Arkema E.V. (2020). Sarcoidosis diagnosis and treatment in Sweden: A register-based assessment of variations by region and calendar period. Respir. Med..

[16] Ungprasert P., Crowson C.S., Achenbach S.J., Carmona E.M., Matteson E.L. (2017). Hospitalization Among Patients with Sarcoidosis: A Population-Based Cohort Study 1987–2015. Lung.

[17] Hutchinson J. (1877). Illustrations of Clinical Surgery.

[18] Gavrysyuk V., Merenkova I., Dziublyk Y., Morska N., Pendalchuk N., Bychenko O., Vlasova N. (2021). Efficacy and tolerability of Methotrexate and Methyprednisolone in Comparative Assessment of Primary and Long-term outcomes in Patients with Sarcoidosis. Diagnostics.

[19] Magombedze G., Pasipanodya J.G., Gumbo T. (2021). Bacterial load slopes represent biomarkers of tuberculosis therapy success, failure, and relapse. Commun. Biol..

[20] Pasipanodya J.G., Smythe W., Merle C.S., Olliaro P.L., Deshpande D., Magombedze G., McIlleron H., Gumbo T. (2018). Artificial intelligence-derived 3-Way Concentration-dependent Antagonism of Gatifloxacin, Pyrazinamide, and Rifampicin During Treatment of Pulmonary Tuberculosis. Clin. Infect. Dis..

[21] Swaminathan S., Pasipanodya J.G., Ramachandran G., Hemanth Kumar A.K., Srivastava S., Deshpande D., Nuermberger E., Gumbo T. (2016). Drug Concentration Thresholds Predictive of Therapy Failure and Death in Children With Tuberculosis: Bread Crumb Trails in Random Forests. Clin. Infect. Dis..

[22] Friedman J., Popescu B.E. (2008). Predictive learning via rule ensembles. Ann. Appl. Stat..

[23] Drake W.P., Culver D.A., Baughman R.P., Judson M.A., Crouser E.D., James W.E., Ayers G.D., Ding T., Abel K., Green A. (2021). Phase II Investigation of the Efficacy of Antimycobacterial Therapy in Chronic Pulmonary Sarcoidosis. Chest.

